# PET|MR Open Source Radiomics Models for Treatment Outcome Prediction in Recurrent GBM

**DOI:** 10.3390/cancers18183008

**Published:** 2026-09-16

**Authors:** Montserrat Carles, Alejandro Mora-Rubio, Tobias Fechter, Sandra Perez-Herrero, Matías Fernández-Patón, Dimos Baltas, Michael Mix, Philipp Meyer, Ilinca Popp, Luis Martí-Bonmatí, Anca L. Grosu

**Affiliations:** 1 Biomedical Imaging Research Group (GIBI230), Health Research Institute La Fe, 46026 Valencia, Spain; 2 Department of Radiation Oncology, Division of Medical Physics, Faculty of Medicine, Medical Center—University of Freiburg, 79106 Freiburg, Germany; 3 Department of Nuclear Medicine, Faculty of Medicine, Medical Center—University of Freiburg, 79106 Freiburg, Germany; 4 Department of Radiation Oncology, Faculty of Medicine, Medical Center—University of Freiburg, 79106 Freiburg, Germany; 5 Department of Radiology, La Fe Polytechnic and University Hospital, 46026 Valencia, Spain

**Keywords:** glioblastoma, PET|MR imaging, radiomics, FET-PET, re-irradiation, treatment outcome and imaging biomarkers

## Abstract

Background/Objectives: Glioblastoma (GBM) is a hard-to-treat cancer with a 5-year survival rate of 5.7% without significant improvement in the past decades. Personalised strategies based on improved tumour characterisation and treatment-outcome prediction could support treatment adaptation and improve therapeutic efficacy. Magnetic Resonance (MR) is the standard imaging modality for GBM radiotherapy (RT) planning. Positron Emission Tomography (PET) with O-(2)-[^18F]fluoroethyl-L-tyrosine (FET) is recommended for distinguishing recurrence from pseudo-progression. However, evidence on PET|MR complementarity for predicting treatment outcome in recurrent GBM remains limited. Methods: We evaluated FET-PET|MR biomarkers to predict Time-To-Progression (TTP), Overall Survival (OS) and Acute Recurrence (AR) in a prospective cohort of 185 recurrent GBM patients from 15 institutions. T1-weighted contrast-enhanced, Fluid-Attenuation Inversion-Recovery, Apparent-Diffusion-Coefficient maps and FET-PET images were analysed. Gross- and Planning-Target Volumes (GTV/PTV) were manually delineated and the intersection of PET|MR-GTVs was defined as MR∩PET. Radiomics models were developed (5-fold cross-validation) and evaluated in a held-out test set. Additionally, nnUNet was used to predict MR∩PET from MR sequences. Results: MR∩PET volume yielded the highest number of statistically significant models, 15 versus 6 for PET|MR-GTVs and 0 for PET|MR-PTVs. Of these 21 models, 19 required the inclusion of PET imaging. The best-performing models discriminated between short and long OS, with *p* < 0.0001 in validation and test, and predicted AR, with ROC-AUC(validation) = 0.81 and AUC(test) = 0.63. By combining both models, poor responders (short OS and/or AR) were identified with Sensitivity = 82% and Specificity = 75%. MR-based prediction of MR∩PET showed a Dice Similarity Coefficient (test) = 0.87 ± 0.12. Conclusions: The PET|MR radiomic models derived from our multicentre prospective cohort support the identification of recurrent GBM patients with poor RT outcomes, potentially enabling future personalised strategies for treatment improvement.

## Simple Summary

Glioblastoma is the most aggressive type of primary brain tumour, and most patients experience tumour regrowth despite surgery, radiotherapy, and chemotherapy. Accurate prediction of treatment outcome is necessary for patient stratification and timely intervention. This study investigated whether combining information from magnetic resonance imaging with amino acid positron emission tomography could improve the prediction of treatment outcomes in patients undergoing repeated radiotherapy for recurrent glioblastoma. By analysing quantitative imaging characteristics from both techniques, we found that the intersection of tumour regions identified by both imaging modalities provided the strongest prognostic information, while positron emission tomography contributed valuable information beyond magnetic resonance imaging alone. These findings support the use of multimodal imaging biomarkers to better identify patients at higher risk of poor outcomes and may contribute to more personalised redefinition of treatment planning in recurrent glioblastoma.

## 1. Introduction

Glioblastoma (GBM) is the most common primary malignant brain tumour, accounting for up to 51.5% of all malignant tumours [1]. Despite a multimodal standard treatment approach, comprising surgical resection, followed by radiation therapy (RT) and chemotherapy, GBM is considered an incurable disease with a median overall survival of nine months [1] and high recurrence rates of over 80% [2]. Currently, RT remains a key therapeutic option for both primary and recurrent tumours [2,3]. Magnetic Resonance (MR) imaging has been traditionally used for diagnosis, staging and treatment planning. In particular, contrast-enhanced T1-weighted (T1wCE) sequences, which offer great anatomical and superior soft-tissue contrast with respect to Computed Tomography (CT), are used for tumour and organs at risk delineation [4,5,6]. However, T1wCE images are influenced by alterations in blood-brain-barrier (BBB) integrity [7,8,9]; caused by previous treatment procedures, complicating the differentiation between true tumour progression and pseudo-progression, especially in recurrent disease. Given these limitations, amino-acid Positron Emission Tomography (PET) has emerged as a valuable complementary tool, in which amino-acid tracers such as O-(2-[18F]fluoroethyl)-L-tyrosine (FET) exploit the increased uptake by tumour cells [8,9]. FET-PET imaging provides metabolic information largely independent of BBB status, and may allow more accurate identification of active tumour tissue and superior discrimination of true progression from treatment-related changes [10,11,12].

Although current ESTRO-EANO guidelines for the delineation of the Gross- and Planning-Target Volumes (GTV/PTV) in recurrent GBM rely primarily on T1wCE and PET [2], other MR sequences may provide complementary information to further characterise the tumour. In particular, T2-weighted Fluid Attenuation Inversion Recovery (FLAIR) can be used to detect areas of tumour infiltration extending beyond enhancing regions on T1wCE [2]. Additionally, some studies suggest that Apparent Diffusion Coefficient (ADC) maps may help assess changes in tumour tissue associated with progression and pseudo-progression [13], as well as tumour response to treatment [14,15]. The integration of complementary information from multiple imaging modalities into RT planning of GBM has been suggested as a means of enabling personalised radiotherapy approaches, potentially enabling dose escalation to clinically relevant tumour subregions [16]. Nevertheless, the identification of high-risk areas remains challenging because the clinical relevance of integrating the complementary information provided by different imaging modalities for defining these regions has not yet been established.

Over the last decade, radiomics [17] has emerged as a powerful tool for extracting quantitative features from medical images to support diagnostic and prognostic assessments [18]. In the context of GBM, radiomic analyses have been applied to predict Overall Survival (OS) and Time to Progression (TTP), among other endpoints. Most studies involved primary disease patients, and radiomic features were extracted from MR sequences, such as studies based on T1wCE images [19,20], or combining multiple sequences [21,22,23]. Beyond conventional MR-based radiomics, some other studies have also investigated the use of other imaging modalities such as FET-PET [24,25], or the combination of both PET and MR [26]. However, in recurrent GBM, where distinguishing true progression from treatment-related changes represents a major clinical challenge, evidence remains limited due to the lack of prospective multicentric studies evaluating multimodal radiomics.

This study included a prospective multicentre cohort of 185 patients with recurrent GBM treated with re-irradiation in the GLIAA trial [8]. The main aim was to provide open-source PET|MR radiomics models for the stratification of recurrent GBM patients according to their predicted RT outcomes. To this end, the cohort was divided into a training set (n = 150) and a held-out test set (n = 35), with the split designed to ensure a balanced distribution of treatment outcomes. First, radiomics models derived from the GTV and PTV on T1wCE and FET-PET images were evaluated. Second, radiomics models derived from the intersection of the T1wCE and FET-PET GTVs (MR∩PET) were evaluated, based on the clinical relevance of regions showing positive tumour findings simultaneously on both modalities. Finally, information from FLAIR and ADC images was incorporated for two purposes: first, to evaluate their performance in radiomics models for RT outcome prediction; and second, to assess their ability, when combined with T1wCE and its GTV, to predict the MR∩PET volume.

## 2. Materials and Methods

### 2.1. Patient Cohort and Available Data

This study included patients from GLIAA (NCT01252459, DRKS00000634, EudraCT No. 2012-001121-27), a prospective, multicentre, randomised clinical trial enrolling patients with histologically confirmed recurrent GBM who had previously undergone radiotherapy. The GLIAA Pilot was conducted prospectively at a single radiation oncology centre between December 2010 and November 2013. The subsequent GLIAA trial was conducted across 15 radiation oncology centres in Germany between November 2013 and August 2021. Of the 185 patients included in the present study, 32 were enrolled in the GLIAA Pilot, 125 were enrolled in the subsequent GLIAA trial at the same centre as the GLIAA Pilot, and 28 were enrolled at the remaining 14 participating centres in Germany.

The re-irradiation protocol for all the patients was defined according to the PTV. All patients underwent stereotactic re-irradiation with a total homogeneous dose of 39 Gy, delivered in 13 fractions of 3 Gy per fraction, once daily, five days per week. The dose was prescribed according to the criteria of the International Commission on Radiation Units and Measurements (ICRU), with at least 95% of the prescribed dose encompassing the PTV. Image-guided radiotherapy was used for treatment delivery, with thermoplastic head immobilisation. Detailed description of procedures and main outcome results have been previously reported [8,9].

The treatment outcomes of interest were:

- **Time to Progression (TTP):** time from the end of radiotherapy treatment to the date of progression. Progression considers the cases when a patient develops a new tumour as confirmed by neuroimaging or dies of a cause directly attributable to GBM. Patients who died for other reasons were censored.
- **Overall Survival (OS):** time from the end of radiotherapy treatment to the death of a patient irrespective of the cause.
- **Acute Recurrence (AR):** considers the cases when a patient develops a new tumour within 90 days, as confirmed by neuroimaging.

Among the 185 patients with available PET|MR imaging and corresponding contours, a test set of 35 patients was defined. Only 5 of the 28 patients enrolled in the remaining 14 participating centres experienced AR. Therefore, we adopted a splitting strategy in which patients were randomly selected within each subgroup to meet the a priori requirement of ensuring a balanced representation of patients with and without AR. This approach was intended to ensure that model performance was evaluated across a wide range of centres, while maintaining a sufficient number of AR events in the held-out test set. The resulting test set included 4 patients with AR and 6 without AR from the GLIAA Pilot; 8 with AR and 10 without AR from the GLIAA trial at the same centre; and 3 with AR and 4 without AR from the remaining 14 participating centres. The training and test sets did not differ significantly in terms of survival distributions (log-rank, *p* > 0.05), with both distributions being consistent with the expected survival of patients with recurrent GBM. Table 1 presents the available clinical variables and outcomes for patients in the training and test sets.

The imaging data available for this cohort included T1wCE, FLAIR, ADC maps and FET-PET/CT exams acquired at the time of recurrence before re-irradiation, with a maximum interval of two weeks between imaging acquisition and treatment initiation. MR scans were acquired across multiple scanners and vendors, including the Siemens TrioTim, Avanto, Prisma, Espree, Biograph, Skyra, and MAGNETOM Vida systems; the GE Signa Explorer; and the Philips Gyroscan Intera and Achieva. Acquisition parameters for the different sequences are presented in Table 2. Anisotropic diffusion filtering [27,28] and the ANTs N4 pipeline for Bias Field Correction [29,30] were applied to T1wCE. PET scans were acquired 20 min after intravenous administration of 3 MBq/kg of FET, with a scan duration of 20 min. FET-PET images were acquired on multiple PET/CT systems, including the Philips Medical Systems GEMINI TF TOF 64, Philips Medical Systems GEMINI TF Big Bore, Philips Vereos PET/CT, and Siemens Biograph scanners. Data were attenuation-corrected using low-dose CT and reconstructed isotropically using iterative reconstruction algorithms (OSEM, 3D-RAMLA, BLOB-OS-TF). The reconstructed PET images had an isotropic spatial resolution of 2 mm^3^. Moreover, the SUV formula employed was ${SUV}_{bw}=\frac{C\left(t\right)}{D_{I}\cdot d}W$, where $C\left(t\right)$ is the activity concentration, $D_{I}$ the injected dose, d the dose decay correction factor, and W the body weight.

A radiation oncologist delineated manually the contrast-enhancing tumour on MR T1wCE, GTV(MR), and delineated semi-automatically the FET uptake areas using a tumour-to-background ratio of 1.8, GTV(PET). The corresponding Planning Target Volume (PTV) was defined with a margin of 4–5 mm in all directions and avoiding critical structures [8]. All contours were defined in accordance with European recommendations [2,3,31]. Additionally, MR∩PET was defined as the spatial intersection of the two GTVs to identify FET-avid regions with corresponding contrast enhancement on T1wCE.

### 2.2. Imaging Modalities and Contours Employed for Radiomic Model Development

**Figure 1:**
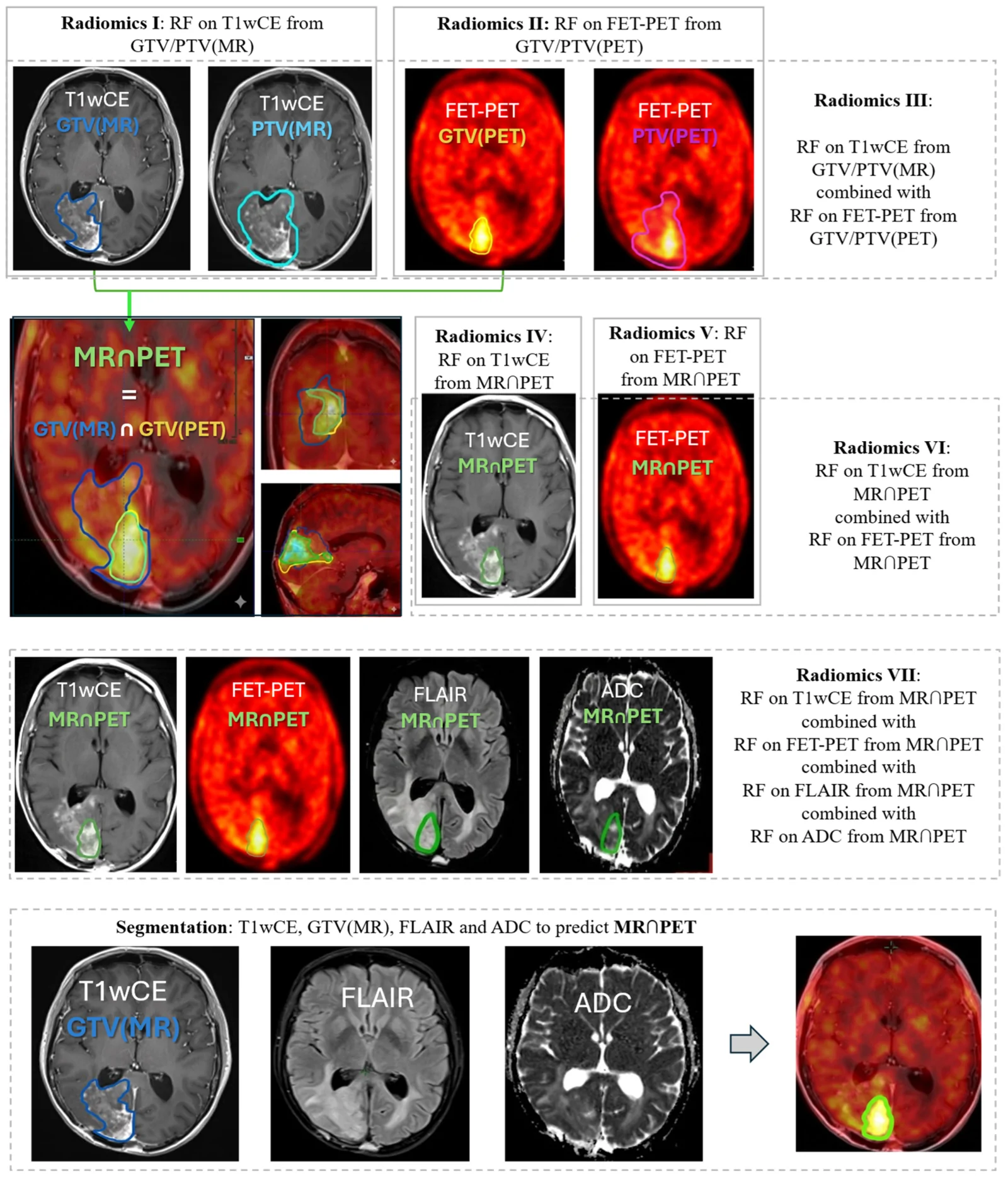
Overview of imaging modalities for each of the radiomics and segmentation analyses.

Figure 1 presents the different approaches evaluated for radiomic model development across imaging modalities and contours:

- T1wCE-GTV/PTV: RF on T1wCE from either the GTV(MR) or PTV(MR) contour.
- PET-GTV/PTV: RF on FET-PET from either the GTV(PET) or PTV(PET) contour.
- T1wCE+PET-GTV/PTV: RF on T1wCE from either the GTV(MR) or PTV(MR) contours combined with RF on FET-PET from either the GTV(PET) or PTV(PET) contour.
- T1wCE-MR∩PET: RF on T1wCE from the intersection between GTV(MR) and GTV(PET).
- PET-MR∩PET: RF on FET-PET from the intersection between GTV(MR) and GTV(PET).
- T1wCE+PET-MR∩PET: RF on T1wCE combined with RF on FET-PET from the intersection between GTV(MR) and GTV(PET).
- Multiparametric MR-MR∩PET with and without PET-MR∩PET: combination of RF on MR T1wCE, RF on T2wFLAIR and RF on ADC maps with and without RF on FET-PET from the intersection between GTV(MR) and GTV(PET).

### 2.3. Radiomic Features Extraction

Radiomics features were extracted using Python 3.11 and PyRadiomics 3.0.1. The pre-processing parameters are presented in Section A of Supplementary Material (Tables SA1–SA4). The RF consisted of 18 first-order statistics features, 14 shape features, 24 features from Grey Level Co-occurrence Matrix (GLCM), 16 from Grey Level Size Zone Matrix (GLSZM), 16 from Grey Level Run Length Matrix (GLRLM), and 14 from Grey Level Dependence Matrix (GLDM). For a detailed description of features and formulas employed, refer to the Image Biomarker Standardisation Initiative [17]. Besides the original image, additional radiomics were derived after image histogram equalisation and wavelet transform, generating a total of 894 features per contour. For contours comprising multiple closed surfaces, the mean RF value was used.

### 2.4. Treatment Outcome Prediction and Performance Evaluation

**Figure 2:**
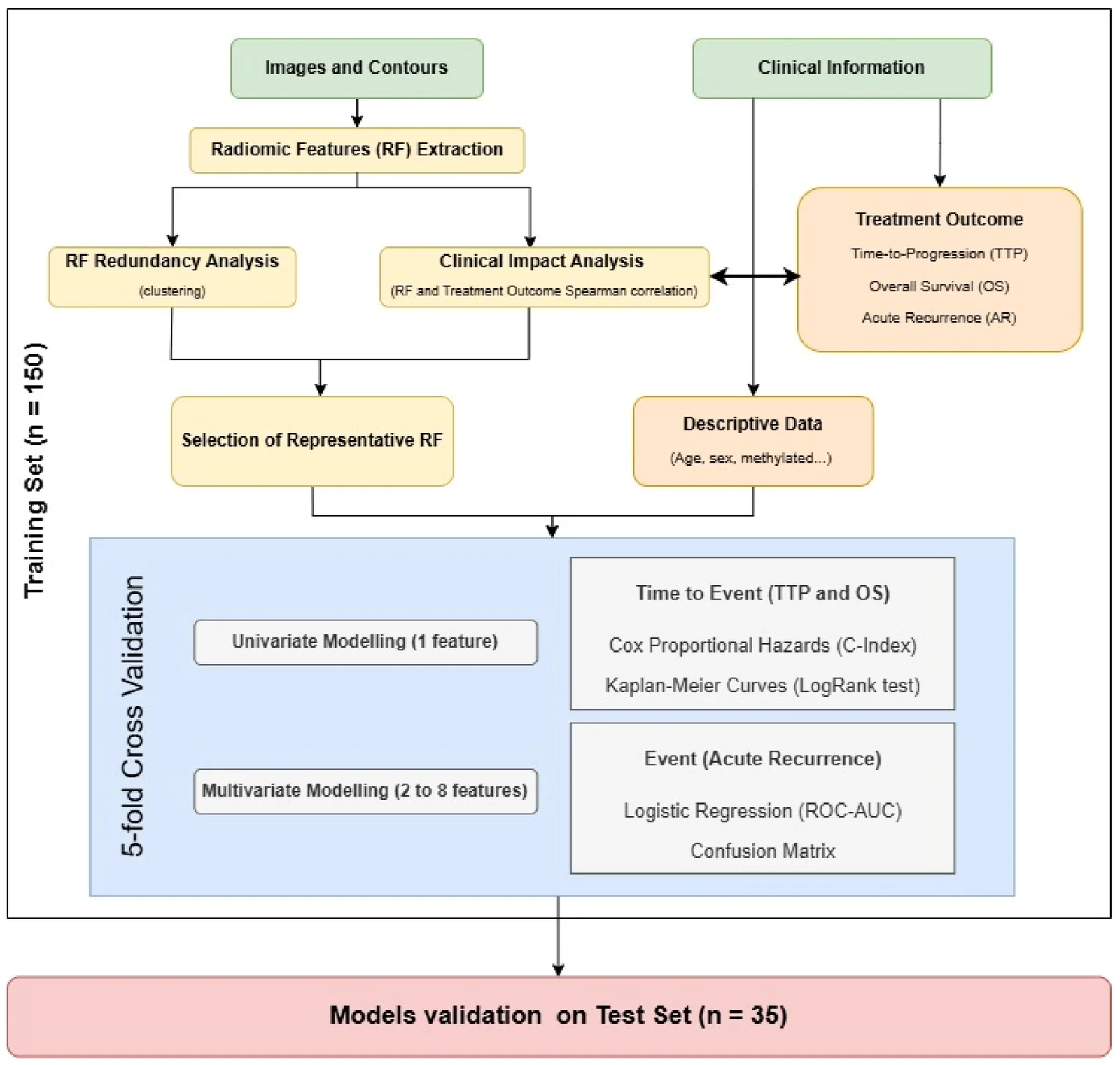
Analysis flowchart for radiomics model development.

Figure 2 presents the analysis flowchart combining clinical information (descriptive and treatment outcome) and radiomics features. The Python 3.11 code implementing the workflow is publicly available at https://github.com/MATTO-GBM-TRANSCAN/Radiomics (accessed on 9 September 2026). First, RFs were extracted from the available imaging data and contours. Second, a redundancy analysis based on clustering was performed to identify groups of highly correlated RFs. In parallel, the clinical impact of each RF was assessed by analysing their Spearman correlations with treatment outcomes (TTP, OS and AR). By combining both analyses, one representative RF with the strongest clinical association was selected from each highly correlated RF cluster for subsequent modelling. Descriptive clinical data were included alongside the representative RFs for further analysis using 5-fold cross-validation.

Both univariate (single feature) and multivariate (signature) analyses were performed. Depending on the clinical endpoint used to assess treatment outcome, two types of tasks were considered: time-to-event tasks (TTP and OS) and event classification tasks (Acute Recurrence).

For the time-to-event tasks (TTP and OS), univariate analysis is evaluated by the LogRank test and Kaplan–Meier curves. For multivariate analysis, a Cox proportional hazards regression model is used to identify the best-performing model prior to Kaplan–Meier analysis, with performance assessed using the Concordance Index (C-index). Outcome prediction in multivariate analysis was considered statistically significant when the following four criteria were simultaneously satisfied: C-Index (5-fold cross-validation) > 0.6, C-Index (test) > 0.6, LogRank *p*-value (test) < 0.05 with the threshold of the median value derived from the training cohort and LogRank *p*-value (test) < 0.05 with the threshold of the median value derived from the test cohort. In univariate analysis, only LogRank was considered. The *p*-values were adjusted for multiple testing using the Benjamini–Hochberg procedure to control the false discovery rate across the representative RFs selected within each outcome/image-contour subgroup.

For the event classification task (Acute Recurrence), the analysis aimed to identify the best classification performance using a logistic regression model, evaluated by the Area Under the Receiver Operating Characteristic Curve (ROC-AUC). Model performance was considered statistically significant for outcome prediction when the following four criteria were simultaneously satisfied: ROC-AUC (5-fold cross-validation) > 0.6, ROC-AUC (test) > 0.6, confusion matrix main diagonal (training) > 0.6 and confusion matrix main diagonal (test) > 0.6, where the main diagonal corresponds to the proportion of true positives and true negatives.

### 2.5. MR Sequences Segmentation Model for Prediction of the Intersection Between GTV(MR) and GTV(PET)

Beyond assessing MR and FET-PET complementarity with regard to treatment outcome prediction, an additional experiment was proposed to evaluate the correlation between the information provided by MR T1wCE, FLAIR, and ADC maps and the regions showing simultaneously FET-avid regions and contrast enhancement on T1wCE (MR∩PET). For this purpose, a segmentation task was formulated (Figure 1), with T1wCE, FLAIR, ADC maps, and GTV(MR) as inputs, and the MR∩PET contour (intersection between GTV(MR) and GTV(PET)) as the target.

The nnU-Net framework [32] was employed. The Plain (no Residual Encoder) 3D full-resolution architecture variant was selected; the batch size was set to 6, the patch size to 128 × 128 × 128, and the number of epochs and folds were set to their default values, based on previous results [33,34].

Model performance was evaluated using the Dice Similarity Coefficient (DSC), Positive Predictive Value (PPV), Sensitivity, and maximum Hausdorff Distance (HD_max_) [35].

## 3. Results

This section presents the main results in three parts. First, radiomic models showing statistically significant predictive performance for treatment outcome are presented, based on images routinely used for RT in recurrent GBM (T1wCE and FET-PET). Second, the complementary value of additional MR sequences is assessed. Finally, the best-performing models are combined to develop a stratification model for poor and good responders, and its accuracy is evaluated in the test cohort.

### 3.1. Models Based on T1wCE and FET-PET

Table 3 summarises the T1wCE and/or FET-PET radiomic models showing statistically significant predictive performance for each evaluated treatment outcome (TTP, OS and AR). For the univariate analysis, the table reports the number of RFs, from each imaging modality and contour, showing a significant association with the outcome. No clinical variable alone showed a statistically significant association with any treatment outcome. For the multivariate analysis, the table indicates whether the resulting signatures included only imaging-derived RFs or a combination of imaging-derived RFs and clinical variables. For each treatment outcome, the combination of image and contour with the model showing the best performance is highlighted in bold, and its performance is detailed in the following. Results of the statistical tests for all other models are presented separately depending on the treatment outcome in the three subsections (TTP, OS and AR) of Section B of the Supplementary Material (Figure SB1, Table SB2 and Table SB3).

The intersection of the GTVs from both modalities (MR∩PET) resulted in the highest number of statistically significant predictive models (N_MR∩PET_ = 15). Among models based on RFs derived from the GTV and PTV, only GTV-derived RFs resulted in statistically significant models (N_GTV_ = 6). Overall, incorporating PET information, either for RF extraction or for MR∩PET definition, resulted in predictive models that provided complementary information to those based on MR alone. Specifically, eight statistically significant models incorporated FET-PET-derived information alone (N_PET_ = 8), while 11 combined FET-PET and T1wCE information (N_PET/T1wCE_ = 11).

The signature comprising RF(T1wCE-GTV) and clinical variables was able to statistically predict TTP with C-Index(5-fold cross-validation) = 0.61 ± 0.03 and C-Index(test) = 0.61. Kaplan–Meier curves and the signature composition are presented in Section B of the Supplementary Materials. The signature showed statistically significant TTP differentiation in Kaplan–Meier curves for the test set: 2.4 vs. 4.9 months (*p* = 0.049) with median derived from the training set and 2.4 vs. 5.1 months (*p* = 0.028) with median derived from the test set.

**Figure 3:**
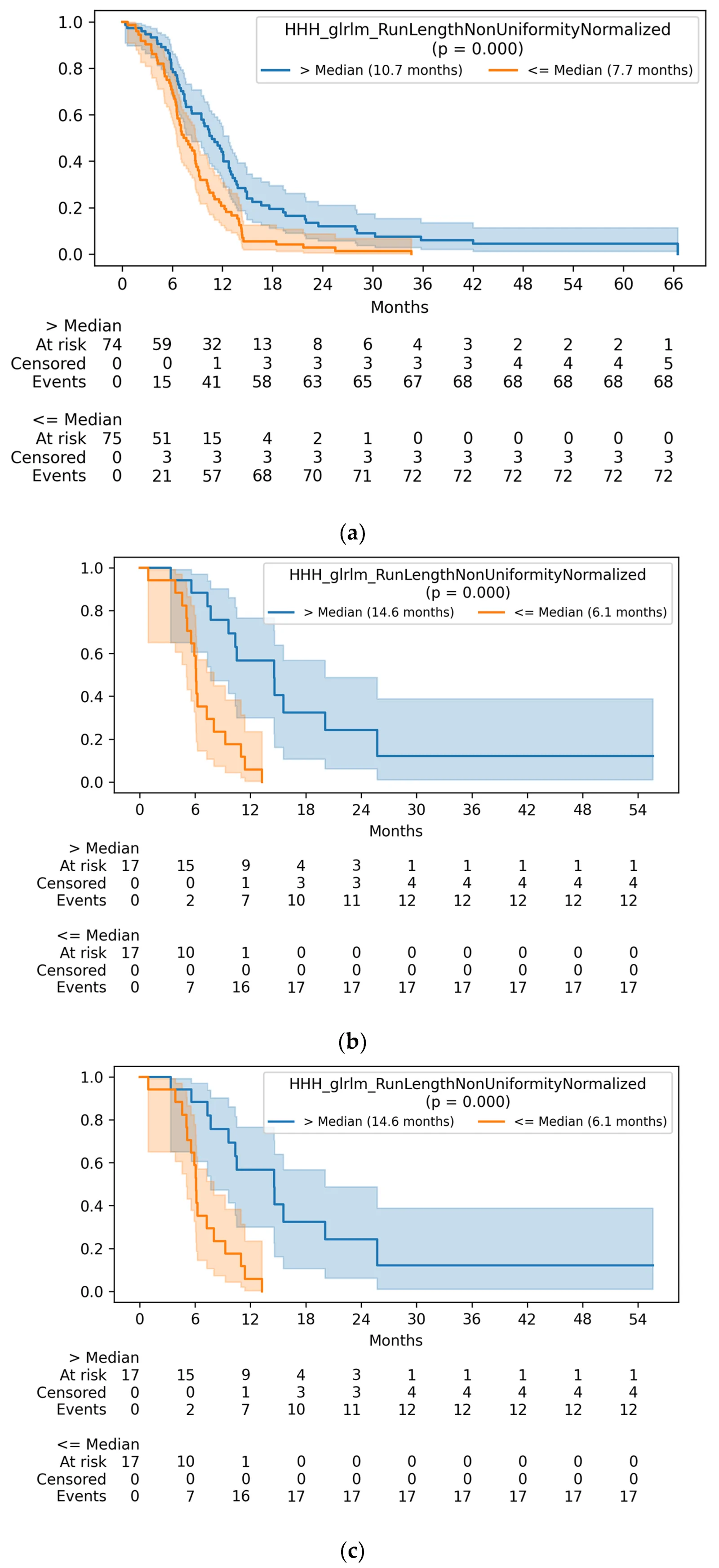
Kaplan–Meier plots for the OS model with glrlm_RunLengthNonUniformityNormalized derived from MR∩PET on Wavelet High-pass filtered in all directions (HHH) T1wCE: (**a**) in 5-fold cross-validation in the training set (C-Index = 0.64 ± 0.04), (**b**) in the test set (C-Index = 0.65) using as threshold the RF median derived from the training set, and (**c**) the median derived from the test set.

Regarding OS, the feature showing the best prognostic performance was glrlm_RunLengthNonUniformityNormalized, extracted from the MR∩PET on Wavelet High-pass filtered in all directions T1wCE (Figure 3). This RF enabled the stratification of patients into short- and long-term OS groups based on whether its value was below or above the median, respectively. In 5-fold cross-validation within the training cohort, the model resulted in a C-Index = 0.64 ± 0.04, and the average values for OS in the differentiated groups were 10.7 and 7.7 months (*p* = 0.000). In the test cohort, the model resulted in C-Index = 0.65 and average OS values of 14.6 vs. 6.1 months (*p* = 0.000), independently of using the median derived from the training set or from the test set (Figure 3b,c).

**Figure 4:**
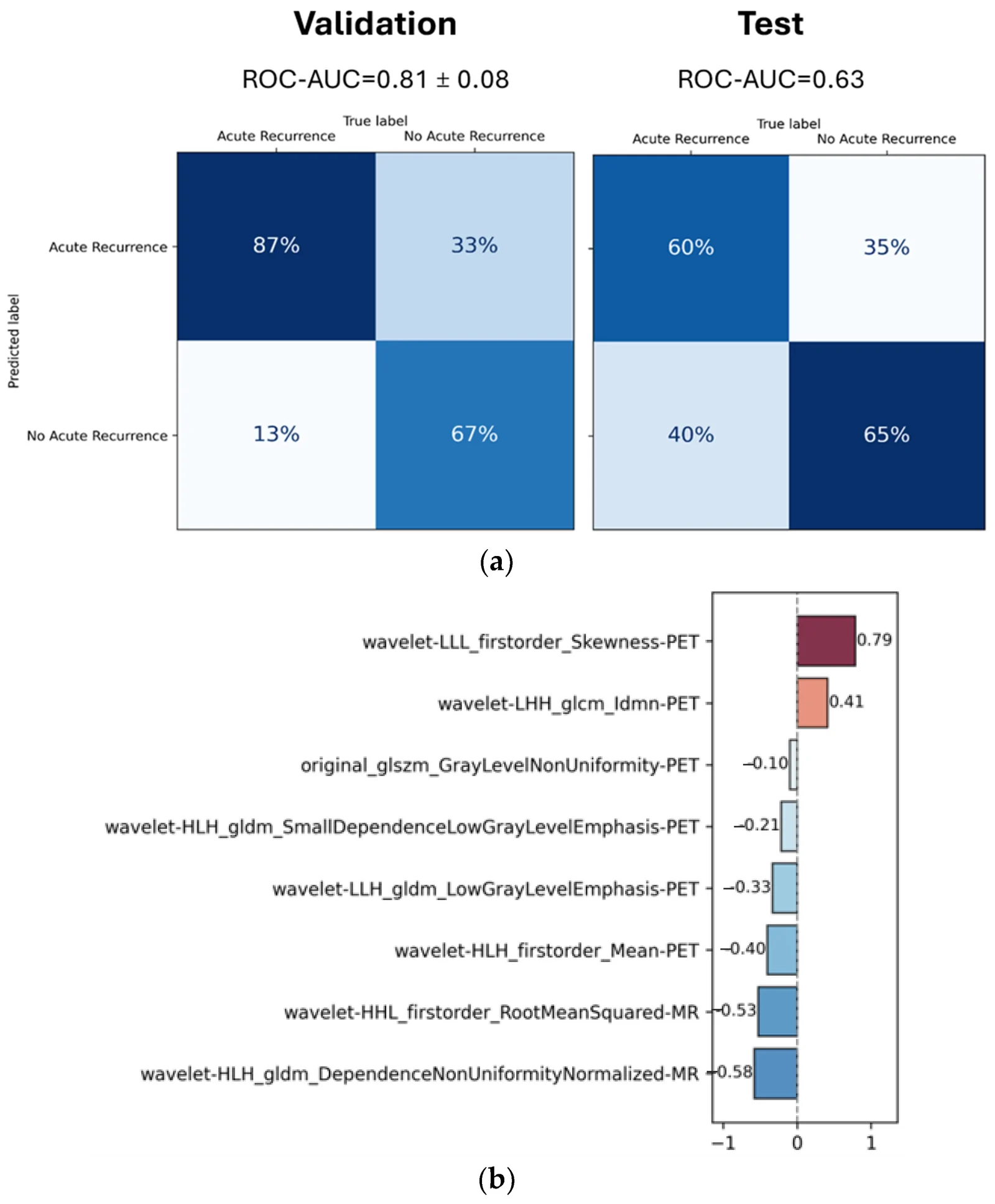
RF(T1wCE+PET-GTV) signature for Acute Recurrence prediction: (**a**) radiomic signature performance based on a column-normalised confusion matrix, with values expressed as percentages and each column summing to 100%; and (**b**) logistic regression coefficients (log-odds scale) of the variables in the signature.

**Figure 5:**
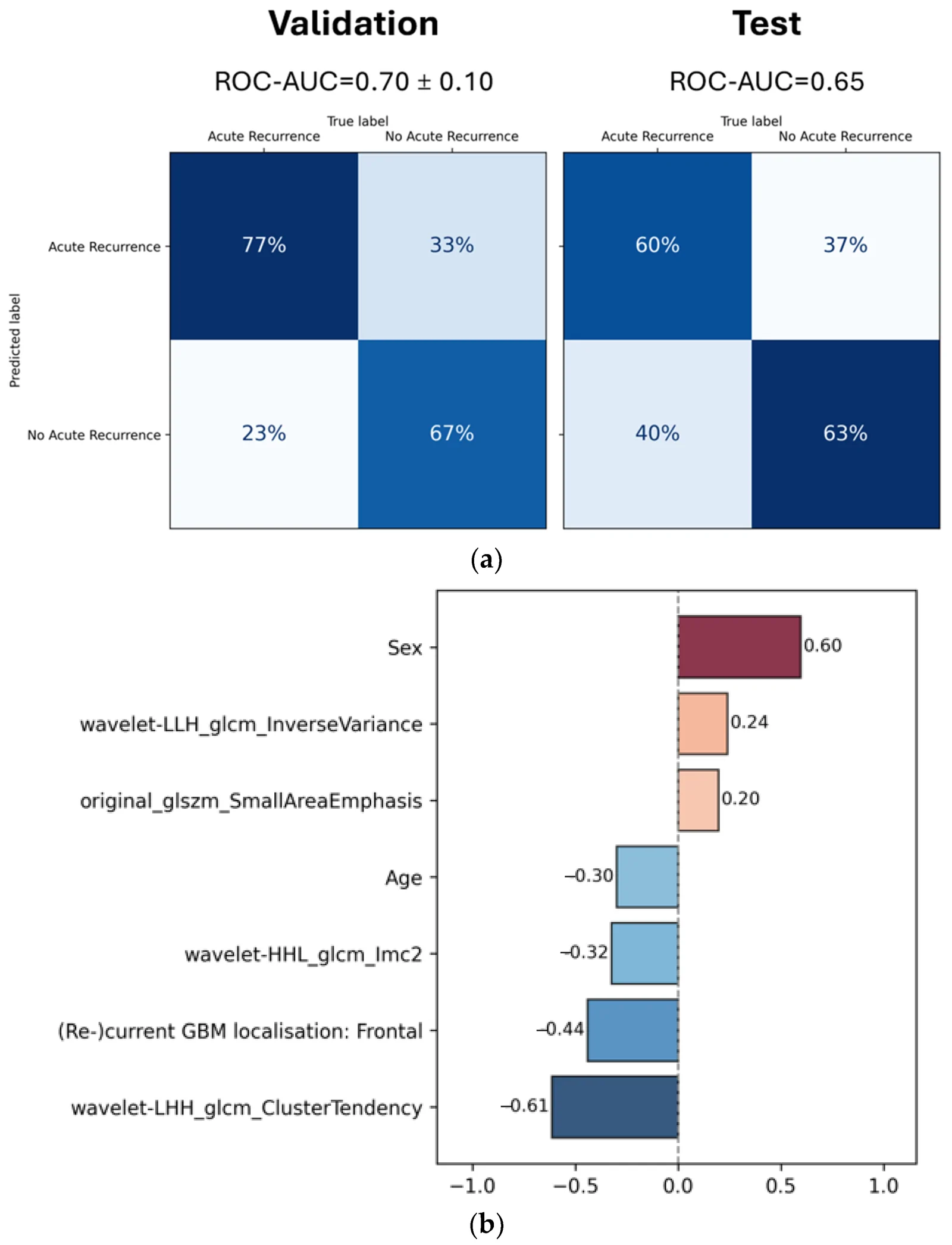
RF(T1wCE-MR∩PET) signature for Acute Recurrence prediction: (**a**) radiomic signature performance based on a column-normalised confusion matrix, with values expressed as percentages and each column summing to 100%; and (**b**) logistic regression coefficients (log-odds scale) of the variables in the signature.

From the models with statistically relevant prediction of AR, two models showed very similar highest performance:

- a combination of RF(T1wCE) and RF(FET-PET) derived from GTV, showing ROC-AUC(validation) = 0.81 ± 0.08 and ROC-AUC(test) = 0.63, in Figure 4, and
- a combination of RF(T1wCE) derived from MR∩PET and clinical variables, showing ROC-AUC(validation) = 0.70 ± 0.10 and ROC-AUC(test) = 0.65, in Figure 5.

The contribution of each variable to the predictive signatures is presented in Figure 4b and Figure 5b. In Figure 4b, the highest contribution (0.79) within the RF(T1wCE+PET-GTV) model corresponds to a histogram feature extracted from the PET image after LLL wavelet filtering. In Figure 5b, the most relevant contributions for the RF(T1wCE-MR∩PET) model (Figure 5) are provided by a texture feature extracted from the MR image after HLH wavelet filtering (−0.61) and the clinical variable gender (0.60), with the latter indicating a higher probability of AR in women.

The best-performing models provided further support for our initial findings, which were based on the number of statistically significant models, regarding the relevance of the GTV and the complementary contribution of FET-PET information. In particular, GTV-derived radiomic features were included in the best-performing models for TTP and AR, whereas FET-PET information contributed either directly, through the extraction of PET radiomic features from the GTV, or indirectly, through its contribution to the definition of the MR∩PET region. Notably, the MR∩PET region was represented among the best-performing models for two of the three treatment outcomes (OS and AR), consistent with its having the highest number of statistically significant predictive models.

In addition, the performance of previously published radiomics models based exclusively on FET-PET imaging [24,25] was assessed in our cohort. However, none of these models achieved statistically significant performance.

### 3.2. Complementarity of FLAIR and ADC

No RF from FLAIR or from the ADC maps, nor a signature containing them, was found to be a significant predictor for any of the treatment outcomes, according to the criteria presented in Section 2.3.

Table 4 summarises the performance of the segmentation model developed to predict the MR∩PET region, using T1wCE, T2wFLAIR, and ADC maps together with the manual GTV(MR) contour as input data.

### 3.3. Patient Stratification Based on Poor Outcomes

The ground truth for poor outcomes was defined as patients exhibiting short overall survival (below the median value of the training set, <262 days) and/or recurrence within 3 months (AR). Performance was evaluated in the held-out test cohort, where 22 patients showed poor outcomes.

Two stratification models for the prediction of patients with poor outcomes were considered, and the results are shown in Table 5. Firstly, the stratification model combined the OS univariate model based on the HHH_glrlm_RunLengthNonUniformityNormalized from the T1wCE -MR∩PET, in Figure 3, and the AR multivariate model with T1wCE + PET- GTV signature, in Figure 4. This model classified poor outcomes in 21 patients; 18 were true positives, but 3 patients showing good outcomes (25%) were classified by the model incorrectly as poor outcomes. Results for the stratification model combining the same OS univariate model with a different AR multivariate model, the T1wCE-MR∩PET signature in Figure 5, predicted 25 patients with poor outcome with 86% of the poor outcomes properly classified, but 50% of the good outcomes incorrectly classified as poor.

## 4. Discussion

In this study, we present PET|MR image-based radiomics models for prediction of RT outcome in patients with recurrent GBM, which have been developed and validated in a multicentric prospective cohort. The methodological approach of this study meets the relevant criteria of the METRICS framework for radiomics research [36] and, in order to facilitate its clinical implementation, the resulting models are available at https://github.com/MATTO-GBM-TRANSCAN/Radiomics (accessed on 9 September 2026).

Our study included radiomics analyses across different volumes of interest (GTV, PTV and MR∩PET). We assumed the number of statistically significant predictive models and the model performance as criteria to assess clinical relevance. Based on our results, the GTV appears to be more relevant than the PTV, which is consistent with expectations, as the GTV represents the tumour-suspected region, whereas the PTV additionally accounts for potential microscopic infiltration and setup uncertainties [3]. Although these uncertainty margins are expected to be small in recurrent glioblastoma, given the need to limit further reirradiation of normal brain tissue already exposed during primary treatment, they may still reduce the strength of the correlation with treatment outcome. These findings are also in agreement with previous FET-PET radiomics analyses of the 32-patient pilot cohort of the GLIAA trial [24], in which the main closed surface with higher uptake demonstrated superior predictive performance compared with analyses considering the entire FET-PET–positive region (SUV > 1.8 × background uptake) with a 3 mm margin.

Additionally, in our evaluation, the intersection of the GTVs derived from T1wCE and from FET-PET (MR∩PET) emerged as the most clinically relevant region for treatment outcome prediction. This observation is in line with previous reports indicating that FET-PET improves the differentiation between true tumour progression and treatment-related changes [8,9]. We developed and validated a segmentation model to predict the MR∩PET region using routinely acquired MR sequences (T1wCE, FLAIR, and ADC). This approach is supported by previous studies demonstrating the value of ADC for distinguishing tumour progression from treatment-related changes [13]. Importantly, such a segmentation model may provide a practical alternative in clinical settings where FET-PET imaging is not available. Nevertheless, these results require extended validation in larger cohorts. In radiomics analysis, however, incorporating FLAIR and ADC radiomics into the predictive models did not provide significant complementary information beyond that obtained from T1wCE radiomics within the MR∩PET region. This suggests that, once the biologically relevant tumour compartment has been accurately identified, conventional contrast-enhanced MR captures most of the imaging information associated with treatment outcome. Overall, these findings further emphasise that accurately distinguishing tumour from treatment-related changes is a critical prerequisite for extracting clinically meaningful radiomic biomarkers capable of predicting treatment outcome.

Regarding outcome prediction, the performance of our radiomics models for TTP, OS, and AR was within the range reported in previous studies on primary glioblastoma, despite the additional complexity of recurrent disease. Our TTP signature (T1wCE) achieved a C-Index 0.61 and *p* = 0.028 in Kaplan–Meier curves, comparable to previously published literature in primary GBM patients reporting for a prospective single-centre cohort (N = 132) a signature (T1wCE) with C-Index = 0.61 [20] and for a retrospective single-centre cohort (N = 274) a signature (T1wCE/FLAIR) with C-Index = 0.68 [23]. Likewise, the univariate OS model resulted from our study achieved a C-index of 0.65 (Kaplan–Meier, *p* < 0.001), only slightly lower than the performance reported for primary GBM patients in a retrospective multicenter cohort (N = 289), where a signature (FLAIR) achieved a C-index of 0.69 [21], and in a retrospective multicenter cohort (N = 865), where a model based on T1wCE, FLAIR, and ADC achieved a C-index of 0.72 [22].

To the best of our knowledge, no previous radiomics studies have specifically addressed the prediction of recurrence within the first three months after treatment in either primary or recurrent glioblastoma. Our two AR signature models achieved AUC-ROC(validation) ≥ 0.70, AUC-ROC(test) ≥ 0.63, and True-Positive and True-Negative ≥ 0.6. Although previous studies have reported AUC values of 0.79 and 0.70 for predicting recurrence in primary glioblastoma [37,38], these studies focused on substantially longer recurrence intervals (7 months and overall recurrence), involved smaller cohort sizes (122 and 129 patients) and did not report the radiomic signatures or detailed classification metrics, limiting direct comparison.

In our study, clinical variables were systematically evaluated across all prediction models. The clinical variables most frequently associated with statistically significant predictions included age, tumour location, MGMT methylation status, and sex. However, the best-performing models did not incorporate these variables. A relevant limitation of the present study is the incomplete availability of isocitrate dehydrogenase (IDH) mutation status across the entire cohort, which limited the assessment of its potential impact on treatment outcome prediction [39]. In the subset of patients for whom this information was available, the inclusion of IDH did not show a statistically significant contribution to either OS or AR prediction models.

An important strength of the present study is the prospective, multicentre design. Although our cohort size (N = 185) was comparable to those reported in the literature, which range from 122 to 865 patients, nearly all previous studies were retrospective and predominantly single-centre. Because endpoints such as TTP, OS, and recurrence strongly depend on accurate longitudinal follow-up, the prospective design reduces uncertainties in outcome definition and increases the reliability of the clinical endpoints. Furthermore, the inclusion of patients from 15 institutions enhances the generalizability of the developed models by reducing centre-specific biases, in contrast to many previous studies in which model development and validation were performed using data from a single institution without multicentric validation. Finally, although previous studies have investigated the role of FET-PET radiomics in both recurrent and primary GBM [24,25], our study enabled a systematic evaluation of the complementary value of MR and FET-PET within the same multi-centre cohort. The complementary information observed for the two imaging modalities in our cohort of 185 patients is consistent with the findings previously reported in a single-centre cohort of 50 patients [11].

In order to facilitate independent validation and future methodological developments, all analysis code has been made openly available. Sharing models and analytical workflows is essential for reproducible and transparent AI research, enabling independent benchmarking, comparison across algorithms, and further assessment of model robustness and generalizability. In this context, we additionally sought to reproduce two previously published FET-PET-based radiomic prognostic models, for which the radiomic features and corresponding thresholds were explicitly reported. These models were derived from cohorts of 32 [24] and 63 [25] patients. This validation is particularly relevant given the more limited availability of FET-PET compared to MR [40,41]. However, neither study’s findings were reproduced in our cohort. For the 32-patient cohort, the discrepancy may reflect both the limited sample size and differences in lesion delineation: in patients with multiple foci, they calculated radiomic features only from the most FET-avid focus, whereas we averaged the values across all components. In our cohort, 60 patients had a single connected component, while the remaining patients had an average number of components of 3 ± 2, ranging from 2 to 11. For the Tumour-Background-Ratio prognostic model derived from the 63-patient cohort [25], our findings are consistent with the lack of prognostic value also reported in another study of 26 patients [42]. This discrepancy may partly reflect differences in imaging timing, as PET was acquired within four weeks after radiotherapy completion in that study, whereas a minimum interval of 9 months after treatment completion was required for inclusion in the GLIAA trial. One of the main limitations of our study is that the underlying clinical dataset cannot currently be released because of data protection constraints. Future work should focus on large PET|MR image repositories to support the development, independent validation, and clinical translation of AI-based PET|MR imaging biomarkers.

Finally, in this study we present a model combining OS and AR prediction for the identification of patients with poor treatment outcomes. Standard treatment has remained almost unchanged for the last decades, and the outcome has not significantly improved (https://seer.cancer.gov). It is apparent that the standard radiation treatment, with homogeneous target definition and dose delivery, is not appropriate to optimise treatment outcomes. Feeding a personalised RT strategy, based on intratumoural heterogeneity, could support strategies focused on the escalation of RT doses to high-risk tumour subareas while sparing doses in organs at risk and thus potentially improving the outcome [16]. The performance of our poor-outcome model was validated in a held-out test set, showing high rates of accuracy and could therefore be suggested for this purpose. In addition, our results support the clinical relevance of combining positive tumour suspicion from both PET|MR imaging (MR∩PET). This region could be integrated into new strategies of dose-escalation, aiming to improve GBM survival rates. Finally, since a segmentation model capable of predicting the MR∩PET volume from standard MR sequences has also been developed, our approach may be applicable both in centres with access to FET-PET imaging and in those without it. Overall, these findings support a step forward in imaging-driven treatment adaptation strategies to address the major therapeutic challenges in GBM.

## 5. Conclusions

A multicentre prospective cohort of 185 patients was used to develop AI-based PET|MR models for treatment outcome prediction. Held-out test validation supports the generalisability of the proposed models, which are publicly available in an open-source repository. The developed poor-outcome model, together with the identification of clinically relevant GBM subregions (MR∩PET), supports the potential of defining alternative, patient-specific treatment strategies aimed at improving survival outcomes in GBM.

## Figures and Tables

**Table 1:** Clinical data from the GLIAA cohort.

	Training Set (150)	Test Set (35)
**Age** (years, median, range)	60 (30–83)	57 (30–77)
**Sex**		
Male	91 (61%)	19 (54%)
Female	59 (39%)	16 (46%)
**GBM**		
Unifocal	77 (51%)	12 (34%)
Multifocal	73 (49%)	22 (63%)
**MGMT status of recurrent tumour**		
Methylated	54 (36%)	11 (31%)
Not methylated	55 (37%)	7 (20%)
Unknown	41 (27%)	17 (49%)
**Surgery before re-irradiation**		
Yes	73 (49%)	22 (63%)
No	77 (51%)	13 (37%)
**Recurrent Tumour Location**		
Right hemisphere	74 (49%)	18 (51%)
Left hemisphere	91 (61%)	18 (51%)
Frontal lobe	62 (41%)	11 (31%)
Parietal lobe	60 (40%)	9 (26%)
Temporal lobe	77 (51%)	19 (54%)
Recurrence/Acute Recurrence *	104/30 (69%/20%)	30/15 (86%/43%)
**Death**	142 (95%)	30 (86%)
**Time to Progression (TTP)** (days, median, range)	118 (9–1336)	102 (28–579)
**Overall Survival (OS)** (days, median, range)	262 (9–1995)	241 (28–1668)

(*) **Acute Recurrence** was defined as the development of a new tumour within 90 days and confirmed by neuroimaging.

**Table 2:** Acquisition parameters for MR sequences.

	Mean ± Standard Deviation (Range)
**Contrast-enhanced T1-weighted (T1wCE)**	
In-plane pixel spacing (mm)	0.7 ± 0.2 (0.2–1.0)
Thickness (mm)	1.0 ± 1.0 (0.9–6.0)
Echo Time (ms)	2.5 ± 0.4 (2.00–3.02)
Flip Angle (°)	14 ± 1 (12–15)
Inversion Time (ms)	940 ± 120 (800–1100)
Repetition Time (ms)	1800 ± 400 (1390–2300)
**T2-weighted Fluid Attenuation Inversion Recovery (FLAIR)**	
In-plane pixel spacing (mm)	0.7 ± 0.2 (0.2–1.0)
Thickness (mm)	4 ± 2 (0.9–6.0)
Echo Time (ms)	170 ± 130 (90–388)
Flip Angle (°)	143 ± 13 (120–180)
Inversion Time (ms)	2300 ± 300 (1800–2719)
Repetition Time (ms)	8000 ± 2000 (5000–11,450)
**Apparent Diffusion Coefficient (ADC) maps**	
b-values (s/mm^2^)	0 and 1000
In-plane pixel spacing (mm)	1.3 ± 0.4 (0.4–1.8)
Thickness (mm)	4.9 ± 0.4 (3.0–6.0)
Echo Time (ms)	91 ± 10 (80–102)
Flip Angle (°)	90
Repetition Time (ms)	3600 ± 500 (3000–5000)

**Table 3:** Number of statistically significant models for Time-to-Progression (TTP), Overall Survival (OS), and Acute Recurrence (AR), according to the imaging modality and contour used for radiomics feature (RF) extraction. Results are reported separately for univariate and multivariate analyses.

	Contour	GTV/PTV			MR∩PET		
	Image	T1wCE	FET-PET	T1wCE + FET-PET	T1wCE	FET-PET	T1wCE + FET-PET
**TTP**	**Univariate**	-	-	-	-	-	-
	**Multivariate**	1 **GTV RF +** **Clinical**	-	-	-	-	-
**OS**	**Univariate**	1 GTV RF	1 GTV RF	-	6 **MR∩PET RF**	6 MR∩PET RF	-
	**Multivariate**		-	1 GTV RF + Clinical	2 MR∩PET RF + Clinical	-	-
**AR**	**Univariate**	-	-	-	-	-	-
	**Multivariate**		1 GTV RF + Clinical	1 **GTV RF**	1 **MR∩PET RF** **+ Clinical**	-	-

For each outcome, models with the best performance are shown in bold.

**Table 4:** Segmentation results for MR∩PET contour.

Set	DSC	PPV	Sensitivity	HD_max_ [mm]
Validation	0.83 ± 0.16	1.00 ± 0.00	0.74 ± 0.20	16 ± 12
Test	0.87 ± 0.12	1.00 ± 0.00	0.79 ± 0.17	14 ± 6

**Table 5:** Performance of the prediction of poor outcomes (OS < 262 d and/or AR) by the models.

Model	Poor Outcomes	True Positive	False Positive
OS+AR(FET-PET+T1wCE)	21	18 (82%)	3 (25%)
OS+AR(T1wCE)	25	19 (86%)	6 (50%)

## Data Availability

The data for the prospective and retrospective cohorts that support the findings of this study were used under licence for the current study and are not publicly available. The source code implemented for this study can be found in the https://github.com/MATTO-GBM-TRANSCAN/Radiomics repository (accessed on 9 September 2026).

## Supplementary Materials

The following supporting information can be downloaded at: https://www.mdpi.com/article/10.3390/cancers18183008/s1, Table SA1: Radiomic Features Extraction Settings for T1wCE images; Table SA2: Radiomic Features Extraction Settings for FET-PET images; Table SA3: Radiomic Features Extraction Settings for FLAIR images; Table SA4: Radiomic Features Extraction ADC maps; Figure SB1: RF(T1wCE-GTV) signature model for TTP prediction: performance by Kaplan-Meier in training (a); in test (b); and RF and Clinical variables contribution (c); Table SB2: Performance of models with statistically significant OS prediction; Table SB3: Performance of models with statistically significant AR prediction.

## Abbreviations

The following abbreviations are used in this manuscript:

BBB	Blood–brain barrier
CNN	Convolutional Neural Network
CT	Computed Tomography
CTV	Clinical Target Volume
DSC	Dice-Sørensen-Coefficient
FET	O-(2)-18F-Fluoroethyl-L-Tyrosine
FLAIR	T2-weighted fluid-attenuated inversion recovery (magnetic resonance sequence)
GBM	Glioblastoma
GT	Ground Truth
HD	Hausdorff Distance
MR	Magnetic Resonance
MR∩PET	MR-Enhancing regions with PET-Uptake overlap
MR-Only	MR-Enhancing regions without PET-Uptake overlap
OARs	Organs at Risk
PC	Prospective Cohort
PET	Positron Emission Tomography
PPV	Positive Predictive Value
PTV	Planning Target Volume
RC	Retrospective Cohort
RF	Radiomic Features
RT	Radiation Therapy
T1wCE	Contrast-enhanced T1-weighted (magnetic resonance sequence)
WSRT	Wilcoxon signed-rank test

## References

[1] Price M., Ballard C., Blakeley J.O., Gondi V., Kruchko C., McCarthy B.J., Van Meir E.G., McNeil D.E., Ryken T.C., Shinojima N. (2024). CBTRUS Statistical Report: Primary Brain and Other Central Nervous System Tumors Diagnosed in the United States in 2017–2021. Neuro-Oncology.

[2] Niyazi M., Andratschke N., Chalmers A.J., Dhermain F., Figini V., Forthuber B., Guha C., Haas-Kogan D.A., Harada T., Iorio-Morin C. (2023). ESTRO-EANO guideline on target delineation and treatment of glioblastoma. Radiother. Oncol..

[3] Andratschke N., Heusel A., Albert N.L., Alongi F., Baumert B.G., Belka C., Castellano A., Dhermain F., Erridge S.C., Grosu A.-L. (2025). ESTRO/EANO recommendation on re-irradiation of recurrent glioblastoma. Radiother. Oncol..

[4] De Pietro G., Di Martino G., Caroprese M., Barillaro A., Cocozza S., Pacelli R., Cuocolo R., Ugga L., Briganti F., Brunetti A. (2024). The role of MRI in radiotherapy planning: A narrative review “from head to toe”. Insights Imaging.

[5] Menze B.H., Jakab A., Bauer S., Kalpathy-Cramer J., Farahani K., Kirby J., Burren Y., Porz N., Slotboom J., Wiest R. (2015). The multimodal brain tumor image segmentation benchmark (BRATS). IEEE Trans. Med. Imaging.

[6] Shukla G., Alexander G.S., Bakas S., Nikam R., Talekar K., Palmer J.D., Shi W. (2017). Advanced magnetic resonance imaging in glioblastoma: A review. Chin. Clin. Oncol..

[7] Brandsma D., van den Bent M.J. (2009). Pseudoprogression and pseudoresponse in the treatment of gliomas. Curr. Opin. Neurol..

[8] Grosu A.-L., Weber W.A., Graf E., Mix M., Nestle U., Schimek-Jasch T., Wiehle R., Mader I., Würtemberger U., Langen K.-J. (2026). Glioma image-guided adaptive radiotherapy (GLIAA): A randomized phase 3 trial. Lancet Oncol..

[9] Oehlke O., Mix M., Graf E., Schimek-Jasch T., Nestle U., Götz I., Schneider-Fuchs S., Weyerbrock A., Mader I., Baumert B.G. (2016). Amino-acid PET versus MRI guided re-irradiation in patients with recurrent glioblastoma multiforme (GLIAA)—Protocol of a randomized phase II trial (NOA 10/ARO 2013-1). BMC Cancer.

[10] Grosu A.-L., Weber W.A. (2010). PET imaging in glioblastoma. Radiother. Oncol..

[11] Lohmann P., Stavrinou P., Lipke K., Bauer E.K., Ceccon G., Werner J.-M., Neumaier B., Fink G.R., Shah N.J., Langen K.-J. (2019). FET PET reveals considerable spatial differences in tumour burden compared to conventional MRI in newly diagnosed glioblastoma. Eur. J. Nucl. Med. Mol. Imaging.

[12] Weber W.A., Grosu A.L., Czernin J. (2008). Technology insight: Advances in molecular imaging and an appraisal of PET/CT scanning. Nat. Clin. Pract. Oncol..

[13] Kamali A., Gandhi A., Nunez L.C., Lugo A.E., Arevalo-Espejo O., Zhu J.-J., Esquenazi-Levy Y., Zhang X., Riascos R.F. (2022). Diffusion MRI-derived apparent diffusion coefficient values for glioblastoma assessment. J. Comput. Assist. Tomogr..

[14] Khalaj K., Jacobs M.A., Zhu J.-J., Esquenazi Y., Hsu S., Tandon N., Akhbardeh A., Zhang X., Riascos R., Kamali A. (2024). Apparent diffusion coefficient as a biomarker of treatment response in glioblastoma patients receiving bevacizumab. Cancers.

[15] Moore-Palhares D., Lawrence L.S., Myrehaug S., Stewart J., Detsky J., Tseng C.-L., Chen H., Dinakaran D., Maralani P., Ruschin M. (2025). Temporal changes in apparent diffusion coefficient predict treatment response in glioblastoma. Int. J. Radiat. Oncol. Biol. Phys..

[16] Brighi C., Keall P.J., Holloway L.C., Walker A., Whelan B., Hamer P.C.d.W., Verburg N., Aly F., Chen C., Koh E.-S. (2022). Dose painting by numbers in glioblastoma: A systematic review. Neurooncol. Adv..

[17] Zwanenburg A., Vallières M., Abdalah M.A., Aerts H.J.W.L., Andrearczyk V., Apte A., Ashrafinia S., Bakas S., Beukinga R.J., Boellaard R. (2020). The Image Biomarker Standardization Initiative: Standardized quantitative radiomics for high-throughput image-based phenotyping. Radiology.

[18] Wisnu Wardhana D.P., Maliawan S., Mahadewa T.G.B., Rosyidi R.M., Wiranata S. (2024). Artificial intelligence and radiomics in glioblastoma: A systematic review. Diagnostics.

[19] Mammadov O., Akkurt B.H., Musigmann M., Ari A.P., Blömer D.A., Kasap D.N.G., Henssen D.J.H.A., Nacul N.G., Sartoretti E., Sartoretti T. (2022). Radiomics for pseudoprogression prediction in high grade gliomas: Added value of MR contrast agent. Heliyon.

[20] Shahzadi I., Seidlitz A., Beuthien-Baumann B., Zwanenburg A., Platzek I., Kotzerke J., Baumann M., Krause M., Troost E.G.C., Löck S. (2024). Residual tumor characterization using PET/MRI radiomics in glioblastoma. Sci. Rep..

[21] Duman A., Sun X., Thomas S., Powell J.R., Spezi E. (2024). Reproducible and Interpretable Machine Learning-Based Radiomic Analysis for Overall Survival Prediction in Glioblas-toma Multiforme. Cancers.

[22] Karabacak M., Patil S., Gersey Z.C., Komotar R.J., Margetis K. (2024). Natural gradient boosting for radiomics-based prediction in glioblastoma. Cancers.

[23] Pak E., Choi K.S., Choi S.H., Park C.-K., Kim T.M., Park S.-H., Lee J.H., Lee S.-T., Hwang I., Yoo R.-E. (2021). DCE MRI radiomics for glioblastoma characterization and prediction. Korean J. Radiol..

[24] Carles M., Popp I., Starke A., Mix M., Urbach H., Schimek-Jasch T., Eckert F., Niyazi M., Baltas D., Grosu A.-L. (2021). FET-PET radiomics in recurrent glioblastoma: Prognostic value for outcome after re-irradiation?. Radiat. Oncol..

[25] Stetter C., Werner J.-M., Wollring M., Ceccon G., Ciantar A., Stoffels G., Mottaghy F.M., Fink G.R., Langen K.-J., Lohmann P. (2025). FET PET radiomics for survival prediction in glioblastoma. Neurooncol. Adv..

[26] Lohmann P., Meißner A.-K., Kocher M., Bauer E.K., Werner J.-M., Fink G.R., Shah N.J., Langen K.-J., Galldiks N. (2021). PET/MRI radiomics in glioblastoma: A multicenter study. Neurooncol. Adv..

[27] Beser-Robles M., Castellá-Malonda J., Martínez-Gironés P.M., Galiana-Bordera A., Ferrer-Lozano J., Ribas-Despuig G., Teruel-Coll R., Cerdá-Alberich L., Martí-Bonmatí L. (2024). Deep learning-based segmentation of glioblastoma in multiparametric MRI. Int. J. Comput. Assist. Radiol. Surg..

[28] Fernández-Patón M., Montoya-Filardi A., Galiana-Bordera A., Martínez-Gironés P.M., Veiga-Canuto D., de Las Heras B.M., Alberich L.C., Solano-Paez P., Martí-Bonmatí L. (2025). Deep learning for diffuse midline glioma classification and segmentation. J. Imaging Inform. Med..

[29] Fernández-Patón M., Alberich L.C., Nebot C.S., Heras B.M.d.L., Canuto D.V., Nieto A.C., Martí-Bonmatí L. (2021). MR image denoising using deep learning for improved brain tumor analysis. J. Digit. Imaging.

[30] Veiga-Canuto D., Fernández-Patón M., Alberich L.C., Pastor A.J., Maya A.G., Sierra J.M.C., Nebot C.S., Heras B.M.d.L., Pötschger U., Taschner-Mandl S. (2024). Reproducibility of radiomics and artificial intelligence methods in neuroblastoma imaging. Radiol. Artif. Intell..

[31] Holzgreve A., Nitschmann A., Büttner M., Schönecker S., Marschner S.N., Fleischmann D.F., Corradini S., Belka C., la Fougère C., Bodensohn R. (2024). FET PET-based target delineation in glioblastoma radiotherapy. Radiother. Oncol..

[32] Isensee F., Jaeger P.F., Kohl S.A.A., Petersen J., Maier-Hein K.H. (2021). nnU-Net: A self-configuring method for deep learning-based biomedical image segmentation. Nat. Methods.

[33] Campos M., Beser-Robles M., Castellá-Malonda J., Martí-Bonmatí L., Mata C., Carles M. (2025). A deep learning approach for brain tumor segmentation using multimodal MRI. Proceedings of the IEEE 38th International Symposium on Computer-Based Medical Systems (CBMS), Madrid, Spain, 18–20 June 2025.

[34] Mora-Rubio A., Kuhn D., Bravo-Vergara C., Fechter T., Mauer M., Beser-Robles M., Radoszynski I.M., Flores R.C., Fostitsch J., Verdú P.G. (2026). Multimodal PET-MR imaging for glioblastoma characterization and radiotherapy planning. Cancer Imaging.

[35] Taha A.A., Hanbury A. (2015). Metrics for evaluating 3D medical image segmentation: Analysis, selection, and tool. BMC Med. Imaging.

[36] Kocak B., Akinci D’Antonoli T., Mercaldo N., Alberich-Bayarri A., Baessler B., Ambrosini I., Andreychenko A.E., Bakas S., Beets-Tan R.G.H., Bressem K.K. (2024). METhodological RadiomICs Score (METRICS): A quality scoring tool for radiomics research endorsed by EuSoMII. Insights Imaging.

[37] Wang J., Yi X., Fu Y., Pang P., Deng H., Tang H., Han Z., Li H., Nie J., Gong G. (2021). Preoperative MRI radiomics for predicting glioblastoma molecular characteristics and prognosis. Front. Oncol..

[38] Liu C., Li Y., Xia X., Wang J., Hu C. (2022). Radiomics-based prediction of glioblastoma recurrence. J. Cancer.

[39] Sipos D., Raposa B.L., Freihat O., Simon M., Mekis N., Cornacchione P., Kovács Á. (2025). Glioblastoma: Clinical Presentation, Multidisciplinary Management, and Long-Term Outcomes. Cancers.

[40] Baid U., Ghodasara S., Bilello M., Mohan S., Calabrese E., Colak E., Farahani K., Kalpathy-Cramer J., Kitamura F.C., Pati S. (2021). The RSNA-ASNR-MICCAI BraTS 2021 benchmark on brain tumor segmentation and radiogenomic classification. arXiv.

[41] Filimonova E., Leone A., Carbone F., Zoli M., Rzaev J., Schukina M., Carretta A., Bianconi A., Cofano F., Morello A. (2026). Glioblastoma MRI Dataset with Standardized Preprocessing, Expert-Validated Segmentation, and MGMT Profiling. Sci. Data.

[42] Henssen D., Rullmann M., Schildan A., Striepe M., Schürer M., Scherlach C., Jähne K., Stassart R., Sabri O., Seidel C. (2026). Dynamic versus static amino acid PET imaging for glioblastoma assessment. Front. Nucl. Med..

