# Supplementary material for: PET|MR Open Source Radiomics Models for Treatment Outcome Prediction in Recurrent GBM
Source: Cancers (Basel). 2026 Sep 16;18(18):3008. doi: 10.3390/cancers18183008 (PMC13605613; doi:10.3390/cancers18183008)
Supplement: Supplementary file 1 [file cancers-18-03008-s001.zip › cancers-4540665-supplementary.pdf]

Supplementary Material

A. Preprocessing

For each image we present a table with the preprocessing parameters.

Table SA1. Radiomic Features Extraction Settings for T1wCE images.

Parameter	Value	Comments
Voxel Dimension	[0.5,0.5,1.0]	Median voxel dimension of the MR T1wCE images in the dataset.
Interpolation	BSpline	
Normalize (Scale)	True (100)	$f(x) = s\frac{x-\mu_x}{\sigma_x}$, s: scale, μ_x: mean, σ_x: std. dev.
Bin Width	GTV(T1wCE): 10<br>PTV(T1wCE): 11	GTV bin numbers ranging from 40 to 120<br>PTV bin numbers ranging from 48 to 126
Voxel Array Shift	300	When normalizing, grey values below the mean will be negative. Shifting by 300 (3 std. dev. * 100) ensures that most voxels are positive.

Table SA2. Radiomic Features Extraction Settings for FET-PET images.

Parameter	Value	Comments
Voxel Dimension	[2,2,2]	Original voxel dimension of the images
Bin Width	0.01	Bin numbers ranging from 23 to 939.

Table SA3. Radiomic Features Extraction Settings for FLAIR images.

Parameter	Value	Comments
Voxel Dimension	-	No resampling was applied.
Interpolation	-	
Normalize (Scale)	True (100)	$f(x) = s\frac{x-\mu_x}{\sigma_x}$, s: scale, μ_x: mean, σ_x: std. dev.
Bin Width	11	Bin numbers ranging from 6 to 120.
Voxel Array Shift	300	When normalizing, grey values below the mean will be negative. Shifting by 300 (3 std. dev. * 100) ensures that most voxels are positive.

Table SA4. Radiomic Features Extraction ADC maps.

Parameter	Value	Comments
Voxel Dimension	-	No resampling was applied.
Interpolation	-	
Normalize (Scale)	True (100)	$f(x) = s\frac{x-\mu_x}{\sigma_x}$, s: scale, μ_x: mean, σ_x: std. dev.
Bin Width	5	Bin numbers ranging from 5 to 128.
Voxel Array Shift	300	When normalizing, grey values below the mean will be negative. Shifting by 300 (3 std. dev. * 100) ensures that most voxels are positive.

B. Performance of radiomics prediction models

In this section we present the performance of the models not detailed in the main document.

B1. TTP

Results for the RF(T1wCE-GTV) signature with statistically significant prediction of TTP are presented in Figure SB1.

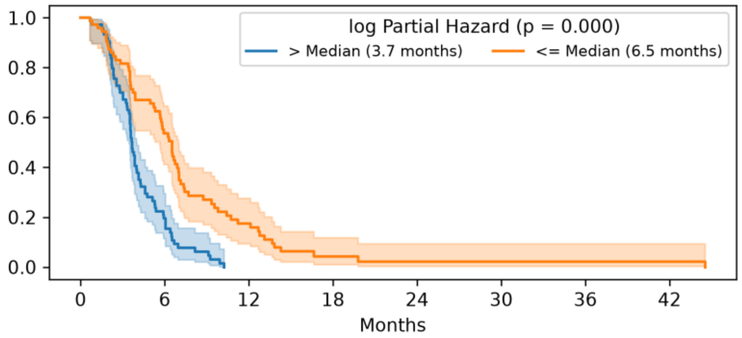

(a) Training set (C-Index= 0.61 ± 0.03)

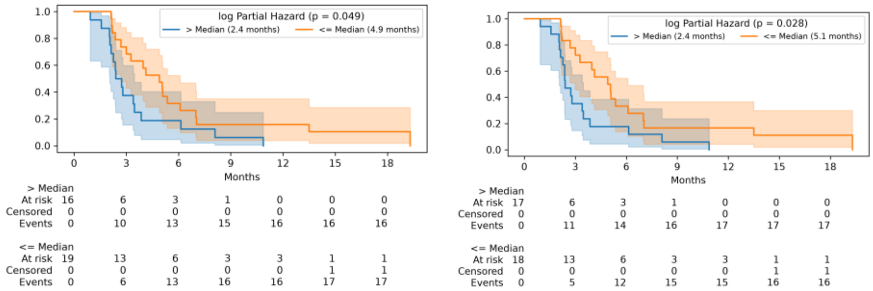

(b) Test set (C-Index= 0.61) with median derived from training set (left) and from test set (right)

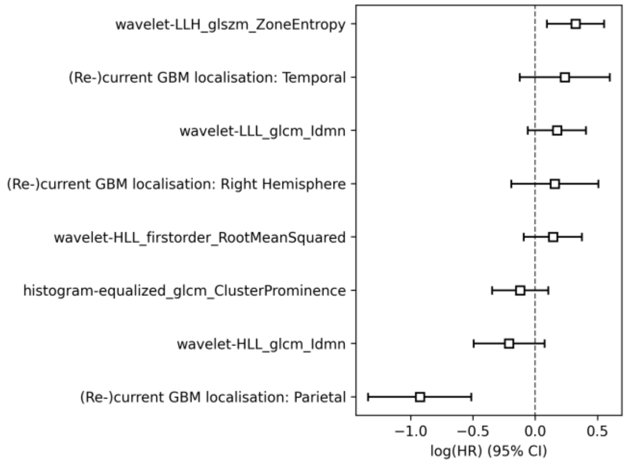

(c)

Figure SB1. RF(T1wCE-GTV) signature model for TTP prediction: performance by Kaplan-Meier in training (a); in test (b); and RF and Clinical variables contribution (c).

B.2. OS

Table SB2. Performance of models with statistically significant OS prediction.

Variable	Median OS [months]	*p*-value

https://doi.org/10.3390/xxxxx

GTV(MR) HHL_gldm_SmallDependenceEmphasis	Train: 11.1 vs. 7.6<br>Test: 11.0 vs. 6.1	Train p = 0.001<br>Test p = 0.002
GTV(PET) wavelet-HHL_gldm_DependenceEntropy	Train: 7.4 vs. 10.4<br>Test: 6.2 vs. 13.3	Train p = 0.001<br>Test p = 0.002
T1wCE -MR∩PET original_glcm_Idmn	Train: 7.4 vs. 10.3<br>Test: 6.1 vs. 10.5	Train p = 0.002<br>Test p = 0.017
T1wCE-MR∩PET HHH_gldm_DependenceNonUniformityNormalized	Train: 7.9 vs. 10.4<br>Test: 7.3 vs. 14.6	Train p = 0.011<br>Test p = 0.009
T1wCE-MR∩PET HHL_gldm_SmallDependenceEmphasis	Train: 10.4 vs. 7.7<br>Test: 14.6 vs. 6.3	Train p = 0.004<br>Test p = 0.002
T1wCE-MR∩PET HLL_gldm_GrayLevelNonUniformity	Train: 7.1 vs. 11.1<br>Test: 6.3 vs. 14.6	Train p = 0.000<br>Test p = 0.027
T1wCE-MR∩PET LLL_gldm_LowGrayLevelEmphasis	Train: 11.1 vs. 7.0<br>Test: 14.6 vs. 7.3	Train p = 0.002<br>Test p = 0.016
FET-PET-MR∩PET histogram-equalized_firstorder_Range	Train: 7.7 vs. 10.7<br>Test: 6.3 vs. 10.5	Train p = 0.001<br>Test p = 0.023
FET-PET-MR∩PET HHH_gldm_DependenceNonUniformityNormalized	Train: 11.1 vs. 7.4<br>Test: 13.3 vs. 6.2	Train p = 0.000<br>Test p = 0.002
FET-PET-MR∩PET histogram-equalized_glszm_ZoneEntropy	Train: 7.4 vs. 11.1<br>Test: 6.3 vs. 10.5	Train p = 0.001<br>Test p = 0.023
FET-PET-MR∩PET LHH_glcm_Idn	Train: 7.4 vs. 10.7<br>Test: 6.3 vs. 14.6	Train p = 0.001<br>Test p = 0.011
FET-PET-MR∩PET HHH_gldm_DependenceVariance	Train: 7.4 vs. 11.4<br>Test: 6.3 vs. 14.6	Train p = 0.000<br>Test p = 0.001
FET-PET-MR∩PET histogram-equalized_gldm_SmallDependenceLowGrayEmphasis	Train: 10.4 vs. 7.9<br>Test: 10.5 vs. 6.3	Train p = 0.005<br>Test p = 0.041

**Signature**	***p*-value test**	**C-Index**	
		**Validation**	**Test**
T1wCE + PET- GTV	0.029	0.68 ± 0.07	0.61
T1wCE -MR∩PET A*	0.000	0.63 ± 0.03	0.75
T1wCE -MR∩PET B *	0.001	0.62 ± 0.02	0.72

(*) signature-A involves 3 RF and age and signature-B involves 4 RF, age, MGMT-status, and localisation in temporal and frontal lobes.

B.3. AR

Table SB3. Performance of models with statistically significant AR prediction.

Signature	ROC-AUC	
	Validation	Test
FET-PET GTV + clinical	0.79 ± 0.06	0.67

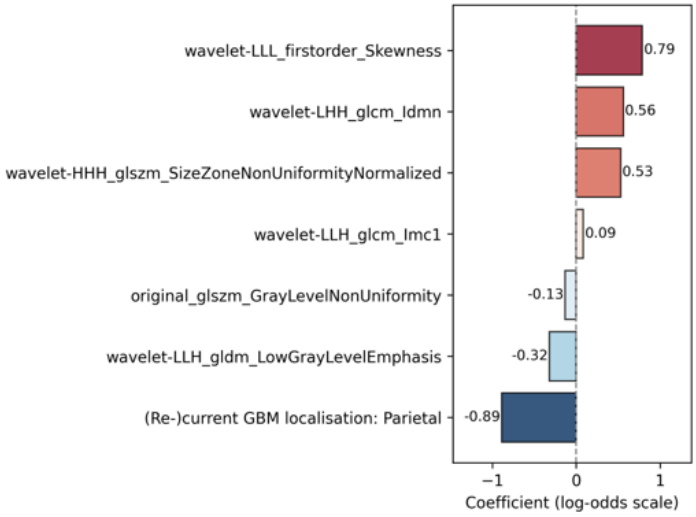

https://doi.org/10.3390/xxxxx
